# Bellini duct carcinoma of the kidney masquerading as an iliac bone tumour in an adult Nigerian

**DOI:** 10.11604/pamj.2017.27.245.12352

**Published:** 2017-08-03

**Authors:** Abdulkadir Ayo Salako, Tajudeen Adeniran Badmus, Innocent Ikem, Kayode Adelusola, Ayodele Elkanah Orimolade, Martin Chukwudum Igbokwe, Kelechi Mc'Clement Onuoha, Foluke Olanike Irabor

**Affiliations:** 1 Urology Unit, Department of Surgery, Obafemi Awolowo University Teaching Hospitals Complex, Ile-Ife, Nigeria; 2 Department of Orthopaedics and Traumatology, Obafemi Awolowo University Teaching Hospitals Complex, Ile-Ife, Nigeria; 3 Department of Morbid Anatomy, Obafemi Awolowo University Teaching Hospitals Complex, Ile-Ife, Nigeria

**Keywords:** Bellini duct carcinoma, iliac bone tumour, Ile-Ife, Nigeria

## Abstract

Bellini Duct Carcinoma (BDC) of the Kidney is a rare type of Renal Cell Carcinoma. It usually presents with features of local advancement or metastasis and rarely diagnosed incidentally. We present a case report of a young man who was found to have BDC of the Right Kidney following presentation with an iliac bone tumour. A 40 year old man presented to the Orthopaedic outpatient clinic on account of right sided pelvic pain and limping following a trivial fall at home. There was no antecedent history of loin pain, loin mass or haematuria. On evaluation, he was found to have a huge right iliac bone tumour invading the contiguous muscles. An incidental hypodense central ipsilateral renal mass with enlarged peri-hilar lymph nodes were found. He subsequently had right radical nephrectomy via a right sub-coastal approach and wide local excision of the Iliac bone tumour in two separate procedures. The resection margins were negative for tumour cells. Histology of the resected specimens were consistent with a metastatic right BDC of the kidney. He had a smooth post-operative recovery. One third of BDC of the kidney presents with metastasis. A high index of suspicion is required in order to diagnose BDC following such unusual presentations.

## Introduction

Renal Cell Carcinoma (RCC) has also been called the "Internist's tumour" due to the myriad of ways it may present [1]. Bellini duct carcinoma (BDC) is a rare type of RCC accounting for less than 3% of Renal Carcinomas [2, 3]. In Nigeria, BDC has been reported as a slightly higher fraction of RCC (4%)by Tijani et al [4]. A 7-year retrospective review of RCC in our center between 2007 and 2014 during which a total of 51 cases of RCC were managed showed no case of BDC [5]. Forty percent (40%) of BDC are known to present with metastatic disease with 66% succumbing to the disease within 2 years of presentation, [6]. It has been found to be more common in men of African descent when compared with Caucasians [7]. The typical clinical presentation is similar to that of clear cell RCC though symptoms from metastatic disease or paraneoplastic syndromes at presentation have been described [8]. These features include gross hematuria, loin pain, loin mass and general weakness [5]. Unusual presentations reported in literature include acute renal failure and cervical lymphadenopathy (Kru Lin et al), multiple osteoblastic bone lesions (Nakamura et al) and meningeal carcinomatosis (Ohnishi et al) [2, 9, 10]. The diagnoses in these cases were not made till autopsy due to the bizzare presentations. We present in this report a 40 year old man with a rare metastatic BDC to the iliac bone managed by the Urology and Orthopaedic Units, Obafemi Awolowo University Teaching Hospital, Ile-Ife, Osun state, Nigeria.

## Patient and observation

**Figure 1:**
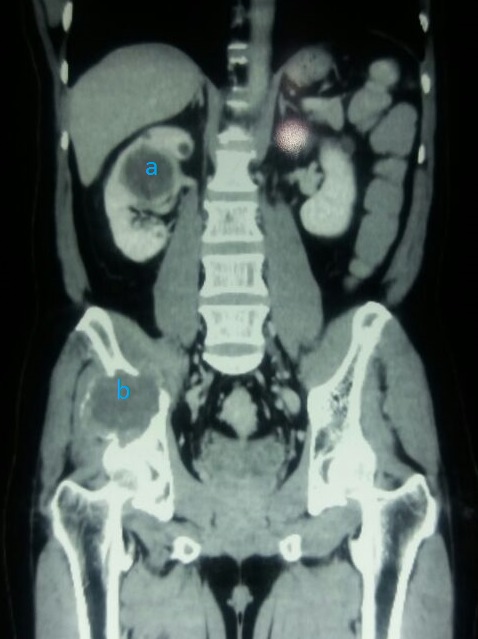
CT scan showing both (A) right renal and (B) pelvic tumours

**Figure 2:**
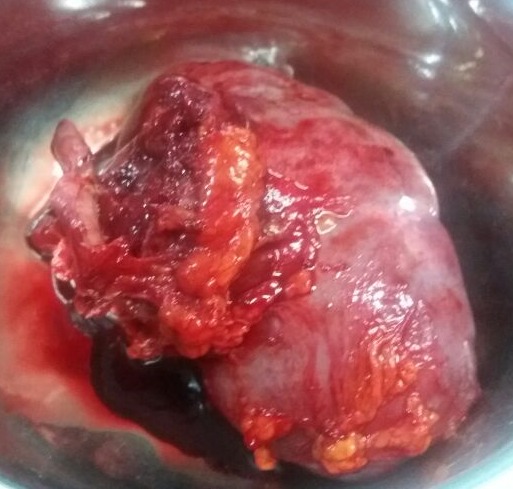
Right kidney with solid mass in the hilar region

**Figure 3:**
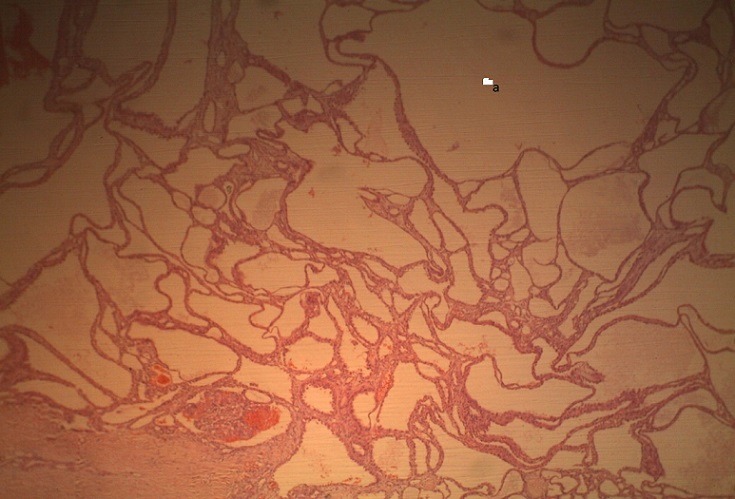
Micrograph showing (A) dilated bellini ducts in the renal medulla

**Figure 4:**
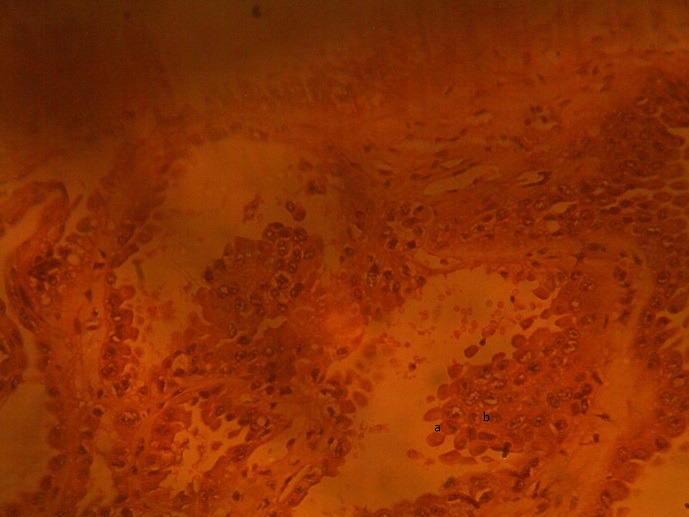
High power micrograph showing (A) hobnail appearance (B) malignant atypical renal epithelial cells

A 40-year-old man presented to the Orthopaedic out-patient clinic on account of a month history of right sided pelvic pain and a limp following a trivial fall at home. There was no preceding history of similar symptoms, haematuria, loin pain or loin mass. There was no weight loss. Initial clinical evaluation was unremarkable. An Abdominopelvic Contrast Computerized Tomography Scan (CT scan) revealed an expansile lytic mass in the right iliac bone, eroding it and invading the contiguous iliacus and gluteal muscles (Figure 1 A). It also showed a large hypodense mass in the central portion of the right kidney, adjacent to the renal hilum with peri-hilar lymph node enlargement (Figure 1 B). The renal vein and inferior vena cava were however not involved.There were no other areas of metastasis. Based on the above findings, a diagnosis of Right RCC (Clinical Stage T3, N1, M1 (Stage 4) was made. He was subsequently scheduled for right radical nephrectomy via a right subcoastal transperitoneal approach. Intra-operatively, we found a solid right renal mass which had invaded the Gerota's fascia in some areas measuring 7 by 8 centimeters. It was resected en bloc with adjoining peri-hilar lymph nodes (Figure 2). Following recuperation, he had wide local excision of the right pelvic mass along with the diseased iliac bone and iliacus muscle through a right Ilio-inguinal approach. Both specimen had negative resection margins. Histopathologic evaluation of the resected kidney specimen revealed proliferation of malignant epithelial cells forming tubules which are cystically dilated. The renal stroma was desmoblastic and infiltrated by some of these malignant cells. Tumour was confined to the gerota's fascia. The findings were consistent with a tubulocystic variant of BDC. Evaluation of the iliac bone specimen was in keeping with a metastasis from BDC of the kidney. The patient made a smooth post-operative recovery and is being planned for commencement of immunotherapy with Sunitinib (Figure 1, Figure 2, Figure 3, Figure 4).

## Discussion

BDC of the kidney also known as collecting duct carcinoma of the Kidney is a rare type of RCC accounting for less that 3% of all cases of RCC and has a tendency for early metastasis and high mortality [2, 3]. Due to these peculiarities, BDC was recognised as a separate entity by the 1998 World Health Organisation (WHO) classification [11]. In our previous report on experience with management of RCC in Ile-Ife, there were no cases of histologically confirmed BDC in over 50 cases owing to its rarity. It is not suprising that the index patient is a 40-year-old male as BDC is commoner in males with a Male: Female ratio of 2:1 and occurs mostly within the age range of 40-71 years [3, 12]. The tumour is also frequently seen in the second and third decades of life, hence in younger patients than conventional RCC [2]. Bloem et al found that majority of bone tumours in patients older than 40 years were malignant [13]. Metastasis, multiple myeloma, chondrosarcoma, Ewing's tumour, osteosarcoma and fibrosarcoma were some of the more common histologic tumours found in this age group [13]. The index patient presented with right sided pelvic pain and a limp following a trivial fall and had none of the clinical triad of loin pain, loin mass or haematuria. This buttresses the fact that the clinical presentation of this tumour can mimic many other clinical conditions, hence requires a high index of suspicion in order to make the diagnosis. The index case displays the importance of thoroughly investigating all suspected metastatic bone tumours in order to establish their primary origin. Harbin et al discussed the role of imaging (CT scan) in making diagnosis of BDC even though there are no pathognomic features [8]. BDC is a pathologic diagnosis [14]. As was seen in the index case, presence of lesions in the medullary region, predominant formation of tubules, desmoplastic stromal reaction, cytologic, high grade malignant cells, infiltrative growth pattern and absence of other typical RCC subtypes strongly suggest BDC [14]. Radical nephrectomy is the surgery of choice for BDC due to the usual central (hilar) location of the tumour and the high propensity of the tumour to invade the collecting sysytem [8]. Several chemotherapeutic and immunotherapeutic regimens have been used for adjuvant treatment in BDC with varying degrees of response. Oudard et al found a 26% remission rate in BDC following use of Gemcitabine + Cisplatin/Carboplatin combination while Tokuda et al in Japan found interferon (IFN) and interleukin-2 (IL-2) to be ineffective [6, 15]. Targetted therapy with sorafenib, sunitinib or temsirolimus seems to be promising [16]. The prognosis of this condition has remained very poor with a 3-year survival as low as 6% for patients with metastatic disease [17]. In our practice, we offer partial or radical nephrectomy to all patients with resectable RCC who can withstand the metabolic response from surgery. Adjuvant immunotherapy with the aforementioned agents are indicated in patients with recurrent or residual disease.

## Conclusion

The presentation of the index patient with an iliac bone mass highlights the bizarre ways in which RCC may present in our clinical practice. BDC is a rare but aggressive subtype of RCC with a poor prognosis. A high index of suspicion with appropriate imaging ,early surgery and histopathological investigations are key in early diagnosis and improving outcome.

## Competing interests

The authors declare no conflict of interest.
